# Supplementary material for: Non-ohmic tissue conduction in cardiac electrophysiology: Upscaling the non-linear voltage-dependent conductance of gap junctions
Source: PLoS Comput Biol. 2020 Feb 25;16(2):e1007232. doi: 10.1371/journal.pcbi.1007232 (PMC7059938; doi:10.1371/journal.pcbi.1007232)
Supplement: S1 Appendix — Details and proofs for the asymptotic formulation and the numerical solution for the NOHM model are presented here. (PDF) [file pcbi.1007232.s001.pdf]

Non-ohmic tissue conduction in cardiac electrophysiology: upscaling the non-linear voltage-dependent conductance of gap junctions

S1 Appendix

Daniel E. Hurtado[1,2,3*], Javiera Jilberto[1,3], Grigory Panasenko[4,5,6]

1 Institute for Biological and Medical Engineering, Schools of Engineering, Medicine and Biological Sciences, Pontificia Universidad Católica de Chile, Santiago, Chile
2 Department of Structural and Geotechnical Engineering, School of Engineering, Pontificia Universidad Católica de Chile, Santiago, Chile
3 Millennium Nucleus for Cardiovascular Magnetic Resonance, Chile
4 Institute Camille JORDAN, Université Jean Monnet, Université de Lyon, Saint-Etienne, France
5 Institute of Applied Mathematics, Vilnius University, Vilnius, Lithuania
6 National Research University "Moscow Power Engineering Institute", Moscow, Russia

* dhurtado@ing.puc.cl

1 Multiscale homogenized model for non-Ohmic conduction

This Appendix contains mathematical and heuristic arguments justifying the multiscale model considered in the central part of the paper and describes the numerical scheme used in the computational experiments. In the first section, we consider the steady-state one-dimensional model of the conduction in a periodic chain of cells separated by weakly conductive thin membrane where gap junctions are embedded. At the microscopic level, the conduction coefficient of the membrane depends on the difference of the potentials at the sides of a membrane. The goal of the multiscale mathematical analysis is the up-scaling of this model and the derivation of a macroscopic non-linear constitutive Ohm's law. This analysis yields an algorithm that relates the microscopic and macroscopic conductivities. The method is based on the homogenization technique (see [1–5] and the bibliography therein). The algorithm is rigorously justified by an estimate for the difference of the exact solution of the one-dimensional conductivity problem and an approximate solution, which is a solution of the homogenized problem. At the end of the first section, we discuss an extension of the upscaled constitutive law to the case of the non-stationary conduction problem. Using heuristic arguments, we suggest a formal generalization of the upscaling procedure in the three-dimensional case. The second section of this Appendix presents a weak formulation for the one-dimensional non-Ohmic homogenization model (NOHM), and the numerical scheme used in the solution of the NOHM.

1.1 Formulation of the non-Ohmic homogenization model and main results

Consider an ε-periodic chain of cells separated by thin membranes of thickness $\varepsilon\delta$, where ε, δ are small positive parameters and consider the conduction of the chain having the conductivity within a cell σ_c of order 1 and conductivity of membranes of order of the small parameter δ nonlinearly depending on the voltage drop at the sides of a membrane. Let $u_{\varepsilon,\delta}$ be the microscopic potential field, and $j_{\varepsilon,\delta}$ be the microscopic current density. We consider the problem of conduction given by

$$-\frac{d}{dx} j_{\varepsilon,\delta}(u_{\varepsilon,\delta}) = 0, \quad x \in (0,1), \tag{1}$$

with boundary conditions

$$j_{\varepsilon,\delta}(u_{\varepsilon,\delta})|_{x=0} = I_0, \quad I_0 \in \mathbb{R}, \quad u_{\varepsilon,\delta}|_{x=0} = 0, \tag{2}$$

where I_0 is an applied current density. Denote the periodically extended space occupied by the cytoplasm by $B^c_{\varepsilon,\delta} = \cup_{k=-\infty}^{\infty}((k+\frac{\delta}{2})\varepsilon, (k+1-\frac{\delta}{2})\varepsilon)$, and the membranes occupy the set of layers $B^f_{\varepsilon,\delta} = \cup_{k=-\infty}^{\infty}((k-\frac{\delta}{2})\varepsilon, (k+\frac{\delta}{2})\varepsilon)$, denote the dilated $1/\varepsilon$ times sets: $B^c_{\delta} = \cup_{k=-\infty}^{\infty}(k+\frac{\delta}{2}, k+1-\frac{\delta}{2})$, and $B^f_{\delta} = \cup_{k=-\infty}^{\infty}(k-\frac{\delta}{2}, k+\frac{\delta}{2})$. Problem (1), (2) is the Neumann's problem for the local Ohm's law given by the following relations

$$j_{\varepsilon,\delta}(u_{\varepsilon,\delta}) = -\sigma_\delta\Big(\frac{x}{\varepsilon}, \{u_{\varepsilon,\delta}\}\Big)\frac{du_{\varepsilon,\delta}}{dx} \tag{3}$$

where the conductivity is described by the following relation

$$\sigma_\delta\Big(\xi, \{u\}\Big) = \begin{cases} \sigma_c, & \xi \in B^c_\delta\ , \\ \\ \delta(1+\mu a(S[u]_{j,\varepsilon}))\sigma_g, & \xi \in (j-\frac{\delta}{2}, j+\frac{\delta}{2})\ , \end{cases} \tag{4}$$

where

$$[u]_{j,\varepsilon} = u((k+\frac{\delta}{2})\varepsilon) - u((k-\frac{\delta}{2})\varepsilon), \tag{5}$$

S is a scaling parameter ($S \le \varepsilon^{-1}$) and a is a smooth function satisfying inequalities

$$\|a\|_{C(\mathbb{R})} \le \bar{a_0}, \|a'\|_{C(\mathbb{R})} \le \bar{a_1}, \tag{6}$$

where $\bar{a_0}, \bar{a_1}, \sigma_c, \sigma_g, \mu$ are positive constants, and ε, δ are small positive parameters. Let us comment the relations (4),(5). The conductivity of a membrane material is small, it is of order of δ compared to the conductivity of the cytoplasm, and it nonlinearly depends on the voltage gap $u((k+\frac{\delta}{2})\varepsilon) - u((k-\frac{\delta}{2})$ scaled by a zooming factor S: $\sigma_\delta = \delta(1+\mu a(S(u((k+\frac{\delta}{2})\varepsilon) - u((k-\frac{\delta}{2}))))\sigma_g$. It means that the conductivity may be sensible to any small voltage gap, i.e. even if the gap is small the variation of the conductivity is significant. The solution of this problem is defined as a continuous piecewise differentiable function, satisfying equations (1),(2).

The main theoretical result of this Appendix concerns the asymptotic analysis of problem (1), (2). We will be prove that there exists a positive constant μ_0 such that for all values of μ such that $|\mu| \le \mu_0$ there exists a unique solution to problem (1), (2), and it can be approximated by a solution v of the homogenized equation

$$\frac{d}{dx}\big(\hat{\sigma}_\delta(\frac{dv}{dx})\frac{dv}{dx}\big) = 0, \quad x \in (0,1), \tag{7}$$

and boundary conditions (same as (2))

$$\hat{\sigma}_\delta(\frac{dv}{dx})\frac{dv}{dx}|_{x=0} = -I_0, \quad I_0 \in \mathbb{R}, \quad v|_{x=0} = 0 \tag{8}$$

where $\hat{\sigma}_\delta(y)$ is the effective conductivity and is given by the following algorithm. First, we solve with respect to the unknown $[N](y)$ the following equation depending on the parameter $y \in [-2(1+\lambda)I, 2(1+\lambda)I]$:

$$[N] = -(1-\delta)\Big(\langle\frac{1}{\sigma_\delta(\cdot, S\varepsilon[N])}\rangle^{-1} - 1\Big)y, \tag{9}$$

where $I = \frac{|I_0|}{\sigma_g}\max\{1,\lambda\}$, $\lambda = \sigma_g/\sigma_c$,

$$\langle F(\cdot)\rangle = \int_0^1 F(\xi)d\xi.$$

Second, we define the effective conductivity tensor

$$\hat{\sigma}_\delta(y) = \langle\frac{1}{\sigma_\delta(\cdot, S\varepsilon[N](y))}\rangle^{-1}. \tag{10}$$

In the following, we will show that for $|\mu| \leq \mu_0$, equation (9) admits a unique smooth solution to problem (7),(8). This problem depends on the small parameter δ, and passing to the limit we can show that the homogenized problem is independent of δ. However we prefer to keep δ in the equation because in this case the error is smaller.

Analysing (9) we can see that if $S\varepsilon$ is a small parameter, then in the case that $a(0) = 0$ using the Taylor's expansion for the function a in the neighborhood of 0 one can prove that the above theorems hold for any finite μ_0. In such case, the contribution of the nonlinearity to the homogenized equation is also relatively small. In the case $S\varepsilon = 1$ the nonlinear effect is strong.

The main theorem proves the error estimates for all μ such that $|\mu| \leq \mu_0$,

$$\|u_{\varepsilon,\delta} - v\|_{L^\infty((0,1))} = O(\varepsilon + \delta^2). \tag{11}$$

Moreover, introducing the first corrector,

$$u^{(1)}_{\varepsilon,\delta} = v + \varepsilon N(\frac{x}{\varepsilon}, v')$$

where $N(\xi, y)$ is a periodic solution to (26), and we obtain

$$\|u_{\varepsilon,\delta} - u^{(1)}_{\varepsilon,\delta}\|_{L^\infty((0,1))} = O(\delta^2), \quad \|\frac{du_{\varepsilon,\delta}}{dx} - \frac{du^{(1)}_{\varepsilon,\delta}}{dx}\|_{L^\infty((0,1))} = O(\delta). \tag{12}$$

These estimates justify the homogenized equation and the effective Ohm's law (10). This law is then used in the numerical simulations on the cable equation in the modeling of electric conductivity of a tissue at the macroscopic scale.

We note that when $\mu = 0$ the membrane conductivity is independent of the transjunctional voltage, which represents an Ohmic behavior of the gap junctions. In such case, there is no need to solve non-linear equation (9), and it is easy to show that the effective conductivity defined in (10) takes on the analytic expression

$$\hat{\sigma}_\delta = \left\{\frac{1}{\sigma_g} + \frac{1-\delta}{\sigma_c}\right\}^{-1} \approx \left\{\frac{1}{\sigma_g} + \frac{1}{\sigma_c}\right\}^{-1}, \tag{13}$$

i.e. the effective conductivity is the harmonic mean of the conductivities of the cytoplasm and of that of the membrane. We note that this is a classical result in linear homogenization theory [1, 2, 5], and corresponds to the classical linear Ohm's law at the macroscopic level. Then, the homogenized problem (7), (8) becomes linear and its solution satisfies (11), while the approximation $u^{(1)}_{\varepsilon,\delta}(x) = v(x) + \varepsilon N(\frac{x}{\varepsilon})v'(x)$ is now the exact solution to the linear version ($\mu = 0$) of problem (1), (2).

1.2 Theorem on the existence and uniqueness of a solution to problem (1), (2)

Theorem 1. *There exists $\mu_0 > 0$ independent of small parameters such that for all $\mu \in [-\mu_0, \mu_0]$ there exists a unique solution $u_{\varepsilon,\delta}$ to problem (1), (2). It satisfies inequality*

$$\|\frac{du_{\varepsilon,\delta}}{dx}\|_{L^\infty((0,1))} \leq \frac{2I}{\delta}. \tag{14}$$

Proof. Denote $U = \frac{du_{\varepsilon,\delta}}{dx}$. Then (1), (2) is equivalent to

$$\begin{array}{l} U(x) = -\frac{I_0}{\sigma_c}, \quad \forall x \in B^c_{\varepsilon,\delta}, \\ U(x) = -\mu a\left(S\int_{(k-\frac{\delta}{2})\varepsilon}^{(k+\frac{\delta}{2})\varepsilon} U(s)ds\right)U(x) - \frac{I_0}{\delta\sigma_g}, \quad \forall x \in ((k-\frac{\delta}{2})\varepsilon, (k+\frac{\delta}{2})\varepsilon), k = 0, \pm 1, ... \end{array} \tag{15}$$

Consider the space $C([(k-\frac{\delta}{2})\varepsilon, (k+\frac{\delta}{2})\varepsilon])$ supplied with the norm

$$\|\cdot\|_E = sup_{x\in[(k-\frac{\delta}{2})\varepsilon, (k+\frac{\delta}{2})\varepsilon]}|\cdot(x)|$$

and a mapping

$$F : E \to E, \quad F(U) = -\mu a \left(S \int_{(k-\frac{\delta}{2})\varepsilon}^{(k+\frac{\delta}{2})\varepsilon} U(s)ds \right) U - \frac{I_0}{\delta \sigma_g}. \tag{16}$$

Note that for any $U_1, U_2 \in E$

$$|F(U_1)(x) - F(U_2)(x)| \leq$$

$$\leq |\mu| |a \left(S \int_{(k-\frac{\delta}{2})\varepsilon}^{(k+\frac{\delta}{2})\varepsilon} U_1(s)ds \right) - a \left(S \int_{(k-\frac{\delta}{2})\varepsilon}^{(k+\frac{\delta}{2})\varepsilon} U_1(s)ds \right) ||U_2(x)| +$$

$$+ |\mu| |a \left(S \int_{(k-\frac{\delta}{2})\varepsilon}^{(k+\frac{\delta}{2})\varepsilon} U_2(s)ds \right) ||U_1(x) - U_2(x)|,$$

and so

$$|F(U_1)(x) - F(U_2)(x)| \leq |\mu| (\bar{a}_1 \delta S \varepsilon \|U_2\|_E + \bar{a}_0) \|U_1 - U_2\|_E. \tag{17}$$

Consider a closed ball $\mathcal{B}_r$ in E with $r = \frac{2|I_0|}{\delta \sigma_g}$. If $|\mu| \leq \frac{1}{2\bar{a}_0}$ then $F(\mathcal{B}_r) \subset \mathcal{B}_r$.

Indeed, if $\|U\|_E \leq r$ then

$$\|F(U)\|_E \leq |\mu| \bar{a}_0 \|U\|_E + \frac{|I_0|}{\delta \sigma_g} \leq \frac{\bar{a}_0}{2\bar{a}_0} \frac{2|I_0|}{\delta \sigma_g} + \frac{|I_0|}{\delta \sigma_g} = r. \tag{18}$$

So, if $U_1, U_2 \in \mathcal{B}_r$ then

$$\|F(U_1)(x) - F(U_2)(x)\|_E \leq |\mu| (\bar{a}_1 \delta S \varepsilon \frac{|2I_0|}{\delta \sigma_g} + \bar{a}_0) \|U_1 - U_2\|_E =$$

$$= |\mu| (2\bar{a}_1 S \varepsilon |I_0| / \sigma_g + \bar{a}_0) \|U_1 - U_2\|_E. \tag{19}$$

Let $|\mu| < \frac{1}{2\bar{a}_1 |I_0| / \sigma_g + \bar{a}_0}$. Then F is a contraction with the factor $\kappa = |\mu| (2\bar{a}_1 |I_0| S \varepsilon / \sigma_g + \bar{a}_0)$ and so there exists a unique fixed point $U \in \mathcal{B}_r$. So, there exists a unique solution such that $\| \frac{du_{\varepsilon\delta}}{dx} \|_{L^\infty((0,1))} \leq \frac{|2I_0|}{\delta \sigma_g} \leq \frac{2I}{\delta}$.

Finally, let us prove that if $|\mu| < \frac{1}{2\bar{a}_0}$ then any solution satisfies this inequality. Indeed, for all $x \in B^c_{\varepsilon,\delta}$ it is evident($\delta < 2$) and for $x \in (0,1) \backslash B^c_{\varepsilon,\delta}$

$$\| \frac{du_{\varepsilon\delta}}{dx} \|_{L^\infty((0,1))} \leq |\mu| \bar{a}_0 \| \frac{du_{\varepsilon\delta}}{dx} \|_{L^\infty((0,1))} + \frac{I}{\delta} < \frac{1}{2} \| \frac{du_{\varepsilon\delta}}{dx} \|_{L^\infty((0,1))} + \frac{I}{\delta}, \tag{20}$$

so,

$$\| \frac{du_{\varepsilon\delta}}{dx} \|_{L^\infty((0,1))} \leq \frac{2I}{\delta}.$$

So, the theorem is proved with

$$\mu_0 < min\{ \frac{1}{2\bar{a}_1 S \varepsilon I + \bar{a}_0}, \frac{1}{2\bar{a}_0} \}. \tag{21}$$

■

In what follows we assume that this condition is satisfied.

1.3 Theorem on the stability of the solution to problem (1), (2)

Theorem 2. *Let condition (6) be satisfied, let f be a piecewise-continous function with possible discontinuities at the points $(k \pm \delta/2)\varepsilon$, i.e.*
$f \in L^\infty(0,1) \cap_{k=0}^{[1/\varepsilon]+1} C([(k-\delta/2)\varepsilon,(k+\delta/2)\varepsilon]\cap[0,1]) \cap C([(k+\delta/2)\varepsilon,(k+1-\delta/2)\varepsilon]\cap[0,1])$ *let $v_{\varepsilon,\delta}$ be a solution to the problem*

$$j_{\varepsilon,\delta}(v_{\varepsilon,\delta})|_{x=0} = I_0 + f, \quad x \in [0,1], \quad v_{\varepsilon,\delta}|_{x=0} = 0 \tag{22}$$

Then there exists $\mu_0 > 0$ independent of small parameters such that for all $\mu \in [-\mu_0, \mu_0]$ the inequality holds

$$\|v_{\varepsilon,\delta} - u_{\varepsilon,\delta}\|_{L^\infty((0,1))} \leq C_{\mu_0}\|f\|_{L^\infty((0,1))}, \tag{23}$$

where C_{μ_0} does not depend on ε, δ.

Proof. Denote $U = \frac{du_{\varepsilon,\delta}}{dx}$, $V = \frac{dv_{\varepsilon,\delta}}{dx}$, then $V - U = -f/\sigma_c$ in $B^c_{\varepsilon,\delta}$ and

$$\|V-U\|_E = \|F(V) - F(U) - \frac{f}{\delta\sigma_g}\|_E \leq |\mu|\bar{a}_1 S\varepsilon\delta\|V-U\|_E\|U\|_E + |\mu|\bar{a}_0\|V-U\|_E + \frac{\|f\|_E}{\delta\sigma_g} \leq$$

$$\leq |\mu|(2\bar{a}_1|I_0|S\varepsilon/\sigma_g + \bar{a}_0)\|V-U\|_E + \frac{\|f\|_E}{\delta\sigma_g}, \quad x \in ((k-\frac{\delta}{2})\varepsilon, (k+\frac{\delta}{2})\varepsilon).$$

Condition (21) yields: $|\mu| < \frac{1}{2\bar{a}_1|I_0 S\varepsilon|/\sigma_g + \bar{a}_0}$, so

$$\|V-U\|_E \leq (1 - |\mu|(2\bar{a}_1|I_0|S\varepsilon/\sigma_g + \bar{a}_0))^{-1}\frac{\|f\|_E}{\delta\sigma_g}$$

for all intervals $[(k-\frac{\delta}{2})\varepsilon, (k+\frac{\delta}{2})\varepsilon]$ and so, integrating $V-U$ and taking into account that the difference $V - U = -\frac{f}{\sigma_c}$ everywhere except for the set $B^f_{\varepsilon,\delta}$ of measure $O(\delta)$, we get:

$$\|v_{\varepsilon,\delta} - u_{\varepsilon,\delta}\|_{L^\infty((0,1))} \leq C_{\mu_0}\|f\|_{L^\infty((0,1))}$$

with $C_{\mu_0} = \max\{(1-\mu_0(2\bar{a}_1|I_0|S\varepsilon/\sigma_g + \bar{a}_0))^{-1}, \lambda\}$. ■

1.4 Derivation of the cell problem

Now consider the homogenization procedure seeking an approximate solution in the form

$$u^{(1)}_{\varepsilon,\delta}(x) = v(x) + \varepsilon N(\frac{x}{\varepsilon}, v'(x)),$$

where v is smooth (belongs to $C^2([0,1])$) and $N(\xi, y)$ is $1-$periodic in ξ, smooth in y. Then

$$-j_{\varepsilon,\delta}(u^{(1)}_{\varepsilon,\delta}) = \begin{cases} \sigma_c(v'(x) + \frac{\partial N}{\partial\xi}(\xi, v'(x)) + \varepsilon\frac{\partial N}{\partial y}(\xi, v'(x))v''(x)), \quad x \in B^c_{\varepsilon,\delta}, \ , \\ \delta\sigma_g(1 + \mu a(S[u^{(1)}_{\varepsilon,\delta}]_{j\varepsilon}))\Big(v'(x) + \frac{\partial N}{\partial\xi}(\xi, v'(x)) + \varepsilon\frac{\partial N}{\partial y}(\xi, v'(x))v''(x)\Big) \ , \\ x \in ((k-\frac{\delta}{2})\varepsilon, (k+\frac{\delta}{2})\varepsilon) \ , \end{cases} \tag{24}$$

where

$$[u^{(1)}_{\varepsilon,\delta}]_{j\varepsilon} = \varepsilon(\delta v'((k+\theta\delta)\varepsilon) + N(k+\frac{\delta}{2}, v'|_{(k+\frac{\delta}{2})\varepsilon}) - N(k-\frac{\delta}{2}, v'|_{(k-\frac{\delta}{2})\varepsilon})) \tag{25}$$

Note that it will be proved that v, v', v'', N and its derivatives are of order of one. Denote $[N](y) = N(k+\frac{\delta}{2}, y) - N(k-\frac{\delta}{2}, y)$. So, keeping the leading term we get the following cell problem: for any real value of a parameter y solve equation

$$\frac{d}{d\xi}\Big(\sigma_\delta(\xi,[N])(\frac{dN}{d\xi}+y)\Big)=0, \tag{26}$$

in the class $H^1_\#$ of 1-periodic functions N belonging to H^1_{loc}, where parameter y stands for $v'(x)$,

$$\sigma_\delta\Big(\xi,[N]\Big)=\begin{cases}\sigma_c, \quad \xi\in B^c_\delta \ ,\\ \\ \delta\sigma_g(1+\mu a(S\varepsilon[N])), \quad \xi\in(k-\frac{\delta}{2},k+\frac{\delta}{2}) \ ,\end{cases} \tag{27}$$

equation (26) is formulated in a classical form, and it is equivalent to the weak formulation: its solution is piecewise linear with respect to ξ. Let us find a 1−periodic solution to (26),(27).

For the leading term of the asymptotic approximation of the current we get:

$$\sigma_\delta\Big(\xi,[N]\Big)(\frac{dN}{d\xi}+y)=C_0=const;$$

$$\frac{dN}{d\xi}=-y+\frac{C_0}{\sigma_\delta\Big(\xi,[N]\Big)}.$$

N is 1−periodic in ξ function and so, $\langle\frac{dN}{d\xi}\rangle=0$ (see Lemma 1 section 3, Chapter 2 from [1]), so,

$$C_0=\langle\frac{1}{\sigma_\delta\Big(\xi,[N]\Big)}\rangle^{-1}y,$$

where $\langle\cdot\rangle=\int_0^1\cdot d\xi$. Note that N is a 1−periodic function, so,

$$[N]=N(\frac{\delta}{2},y)-N(1-\frac{\delta}{2},y)=-\int_{\frac{\delta}{2}}^{1-\frac{\delta}{2}}(-y+C_0/\sigma_c)d\xi$$

because for $\xi\in(\frac{\delta}{2},1-\frac{\delta}{2})$, $\sigma_\delta\Big(\xi,[N]\Big)=\sigma_c$.

So, we get the main equation for unknown function $[N](y)$:

$$[N](y)=-(1-\delta)\Big(\langle\frac{1}{\sigma_\delta\Big(\xi,[N]\Big)}\rangle^{-1}\sigma_c^{-1}-1\Big)y \tag{28}$$

Let us rewrite it calculating the right-hand side.

$$\langle\frac{1}{\sigma_\delta\Big(\xi,[N]\Big)}\rangle^{-1}=(\frac{\delta}{\delta(1+\mu a(S\varepsilon[N]))\sigma_g}+\frac{1-\delta}{\sigma_c})^{-1}=\sigma_g(\frac{1}{1+\lambda}+b_\delta(S\varepsilon[N])),$$

where

$$b_\delta(S\varepsilon[N])=\frac{(1+\delta\lambda)\mu a(S\varepsilon[N])+\delta\lambda}{(1+\lambda)(1+(1-\delta)(1+\mu a(S\varepsilon[N]))\lambda)}; \tag{29}$$

here if $\delta<1$, $|\mu|<\frac{1}{2\bar a_0}$ and so

$$|b_\delta(S\varepsilon[N])|\le\frac{(1+\delta\lambda)|\mu|\bar a_0+\delta\lambda}{1+\lambda}.$$

Equation (28) has the form

$$[N](y)=\varphi(S\varepsilon[N],y), \tag{30}$$

where

$$\varphi(t,y)=-(1-\delta)(\lambda b_\delta(t)-\frac{1}{1+\lambda})y. \tag{31}$$

We will prove further that this mapping is contracting.

1.5 Homogenized equation

Conformly to the homogenization theory for a stratified medium, the homogenized equation can be presented in the form

$$\frac{d}{dx}\Big(\langle\frac{1}{\sigma_\delta\Big(\xi,[N](v')\Big)}\rangle^{-1}v'\Big)=0,\quad x\in(0,1), \tag{32}$$

so that

$$\langle\frac{1}{\sigma_\delta\Big(\xi,[N](v')\Big)}\rangle^{-1}v'=-I_0,\quad x\in(0,1),\quad v(0)=0, \tag{33}$$

i.e.

$$\frac{1}{1+\lambda}v'+b_\delta(S\varepsilon[N](v'))v'=-I_0/\sigma_g,\quad x\in(0,1),\quad v(0)=0. \tag{34}$$

If

$$0<\frac{(1+\delta\lambda)|\mu|\bar{a}_0+\delta\lambda}{1+\lambda}<\frac{1}{2(1+\lambda)}, \tag{35}$$

then $|b_\delta(t)|<\frac{1}{2(1+\lambda)}$, consequently, $|v'|<2(1+\lambda)I$, and so equation (28) should be solved only for values of the parameter $y\in(-2(1+\lambda)I,2(1+\lambda)I)$. Condition (35) is satisfied in particular if

$$\mu_0<\frac{1}{8\bar{a}_0},\delta<\frac{1}{8\lambda}. \tag{36}$$

Below we assume that this condition is satisfied.

1.6 Proof of the existence and uniqueness of a solution to the cell problem

Let us prove that there exists μ_0 independent of δ such that for all $\mu\in(-\mu_0,\mu_0)$ there exists a unique solution $[N]$ to (28), differentiable with respect to y. Let us calculate

$$(b_\delta(t))'=\frac{\mu a'(t)}{(1+(1-\delta)(1+\mu a(t))\lambda)^2}.$$

For μ_0,δ satisfying (36) we have an estimate:

$$|(b_\delta(t))'|<\mu_0\bar{a}_1. \tag{37}$$

There exists μ_0 depending on $\bar{a}_0$ and on $\bar{a}_1$ such that for $\mu\in(-\mu_0,\mu_0)$,

$$S\varepsilon|(b_\delta(t))'|<\frac{1}{2I(1+\lambda)^2}. \tag{38}$$

In particular, it is satisfied if

$$\mu_0<\frac{1}{2I\bar{a}_1S\varepsilon(1+\lambda)^2} \tag{39}$$

and (36).

Then mapping φ from $\mathbb{R}$ to $\mathbb{R}$, defined by the right hand side of relation (30) is a contraction with the factor $\frac{\lambda}{1+\lambda}$ in the interval $[-2(1+\lambda)I,2(1+\lambda)I]$. So there exists a unique solution to equation (28). According to the implicit function theorem, $[N]$ is differentiable on $[-2(1+\lambda)I,2(1+\lambda)I]$.

1.7 Theorem on the existence and uniqueness of a solution to the homogenized problem. Error estimate.

Theorem 3. *There exists $\mu_0 > 0$ independent of small parameters such that for all $\mu \in (-\mu_0, \mu_0)$, there exists a unique solution v of problem (32), (33) (i.e. of the equivalent equation (34)) and a unique solution $u_{\varepsilon,\delta}$ of problem (1), (2) and the following estimate holds*

$$\|v - u_{\varepsilon,\delta}\|_{L^\infty((0,1))} = O(\varepsilon + \delta^2). \tag{40}$$

Proof. Let us prove first the existence of a solution to problem (34) for the values of $\mu \in (-\mu_0, \mu_0)$ such that

$$\|b_\delta\|_{L^\infty(\mathbb{R})} + \|b'_\delta\|_{L^\infty(\mathbb{R})} S\varepsilon \|[N]'\|_{L^\infty([-2(1+\lambda)I, 2(1+\lambda)I])} 2(1+\lambda)I < \frac{1}{2(1+\lambda)}.$$

Let us give a sufficient condition for it using the previously obtained bounds (36), (37), (39). Note that for $[N]'(y)$ we get the following equation:

$$[N]'(y) = -(1-\delta)\Big(\langle \frac{1}{\sigma_\delta\Big(\cdot, [N](y)\Big)}\rangle^{-1}\sigma_c^{-1} - 1\Big) - (1-\delta)\sigma_c^{-1}\Big(\langle \frac{1}{\sigma_\delta\Big(\cdot, [N]\Big)}\rangle^{-1}\Big)'_{[N]}[N]'(y)y =$$

$$= -(1-\delta)\lambda b_\delta(S\varepsilon[N]) + (1-\delta)\frac{1}{1+\lambda} - (1-\delta)\lambda b'(S\varepsilon[N])S\varepsilon[N]'(y)y.$$

Let $\mu_0 < \min\{\frac{1}{32\bar{a}_0}, \frac{1}{32\lambda\bar{a}_0}, \frac{1}{32\bar{a}_1 IS\varepsilon(1+\lambda)^2}\}$ and let $\delta < \min\{\frac{1}{32}, \frac{1}{32\lambda}\}$, then

$$|b_\delta| \le \frac{3}{16(1+\lambda)},$$

$$|b'_\delta| \le |\mu|\bar{a}_1 \le \frac{1}{32IS\varepsilon(1+\lambda)^2},$$

and so, we get

$$|[N]'| \le \frac{3\lambda}{16(1+\lambda)} + \frac{1}{1+\lambda} + \lambda|\mu|\bar{a}_1|[N]'|2S\varepsilon(1+\lambda)I \le$$

$$\le \frac{3\lambda+16}{16(\lambda+1)} + \frac{\lambda}{16(1+\lambda)}|[N]'|.$$

So, $|[N]'|$ is bounded by $\frac{3\lambda+16}{15\lambda+16}$.

So,

$$\|b_\delta\|_{L^\infty(\mathbb{R})} + \|b'_\delta\|_{L^\infty(\mathbb{R})} S\varepsilon \|[N]'\|_{L^\infty([-2(1+\lambda)I, 2(1+\lambda)I])} 2(1+\lambda)I$$
$$\le \frac{3}{16(1+\lambda)} + \frac{3\lambda+16}{32(1+\lambda)^2 I(16+15\lambda)} 2(\lambda+1)I < \frac{1}{2}.$$

So, existence and uniqueness of solution to equation (34) follows from the Banach fixed point theorem applied to the space $[-2(1+\lambda)I, 2(1+\lambda)I]$.

The existence and uniqueness of solution to equation (1), (2) follows from Theorem 1.

Evidently, v is linear, so, $v'' = 0$.

So,

$$-j_{\varepsilon\delta}(u^{(1)}_{\varepsilon\delta}) = \sigma_\delta(\frac{x}{\varepsilon}, [N](y))(\frac{dN}{d\xi} + y)|_{y=v'} + R_{\varepsilon\delta}(x) =$$

$$= \langle \frac{1}{\sigma_\delta\Big(\cdot, [N]\Big)}\rangle^{-1} v'(x) + R_{\varepsilon\delta}(x) = -I_0 + R_{\varepsilon\delta}(x),$$

$$R_{\varepsilon\delta}(x) = (-\sigma_\delta(\frac{x}{\varepsilon}, [N](y)) + \sigma_\delta(\frac{x}{\varepsilon}, \{u^{(1)}_{\varepsilon\delta}\}))(\frac{dN}{d\xi} + y)|_{y=v'} =$$

$$= \begin{cases} 0, x \in B^c_{\varepsilon,\delta} \ , \\ \\ \delta\mu(a(S[u^{(1)}_{\varepsilon,\delta}]_{j\varepsilon}) - a(S\varepsilon[N](v')))(v' + \frac{dN}{d\xi}(v')), \quad x \in ((j-\frac{\delta}{2})\varepsilon, (j+\frac{\delta}{2})\varepsilon) \ , \end{cases} \tag{41}$$

Here

$$|a(S[u^{(1)}_{\varepsilon,\delta}]_{j\varepsilon}) - a(S\varepsilon[N](v')| \leq \bar{a}_1 |S[u^{(1)}_{\varepsilon,\delta}]_{j\varepsilon} - S\varepsilon[N](v')|.$$

Using relation (25), we see that this right-hand side is less than $\bar{a}_1\delta|v'| \leq 2(1+\lambda)\delta I \bar{a}_1$.

So,

$$\|\frac{du^{(1)}_{\varepsilon,\delta}}{dx} - \frac{du_{\varepsilon,\delta}}{dx}\|_{L^\infty((0,1))} = O(\delta), \quad \|u^{(1)}_{\varepsilon,\delta} - u_{\varepsilon,\delta}\|_{L^\infty((0,1))} = O(\delta^2) \tag{42}$$

according to Theorem 2. This estimate justifies the homogenized equation and respectively the nonlinear macroscopic Ohm's law. Theorem 3 is proved. ■

1.8 Extensions to the transient problem of conduction and to three-dimensional domains

Further this law is used for numerical computations of the cable equation at the macroscopic scale.

Remark 1. Theorems 1-3 can be easily generalized in the case when equation (1) is non-homogeneous:

$$-\frac{d}{dx} j_{\varepsilon,\delta}(u_{\varepsilon,\delta}) = f(x), \quad x \in (0,1), \tag{43}$$

where f is a continuous function in $[0,1]$. However this generalization needs several modifications and some additional estimates. Namely, $|I_0|$ in the estimates should be replaced by $I'_0 = |I_0| + \int_0^1 |f(x)|dx$ and the constant I should be replaced by I', obtained from I replacing $|I_0|$ by I'_0. Equation (32) will have the right-hand side $f(x)$:

$$\frac{d}{dx}\Big(\langle \frac{1}{\sigma_\delta\Big(\xi, [N](v')\Big)} \rangle^{-1} v'\Big) = f(x), \quad x \in (0,1), \tag{44}$$

and consequently, (34) will have the following form:

$$\frac{1}{1+\lambda} v' + b_\delta(S\varepsilon[N](v'))v' = -I_0/\sigma_g + \int_0^x f(\theta)d\theta, \quad x \in (0,1), \quad v(0) = 0. \tag{45}$$

By the implicit function theorem, v' is continuously differentiable and it is no more constant. Finally, (40) will have an additional residual consisting of two parts.The first one is

$$\delta(1 + \mu a(S[u_{\varepsilon,\delta}]_{j\varepsilon})\varepsilon \frac{\partial N}{\partial y}(\xi, v'(x))v''(x)$$

and the second one is related to the approximation

$$\delta\mu a(S[u^{(1)}_{\varepsilon,\delta}]_{j\varepsilon}) = \delta\mu a(S\varepsilon(N(j+\frac{\delta}{2}, v'(j\varepsilon)) - N(j-\frac{\delta}{2}, v'(j\varepsilon))) + r_{\varepsilon,\delta},$$

$$|r_{\varepsilon,\delta}| \leq \bar{a}_1\delta\mu(\delta \sup_{x\in[0,1]} |v'(x)| + \frac{\delta\varepsilon}{2} \sup_{\xi\in[0,1], y\in[-2(\lambda+1)I', 2(\lambda+1)I']} |\frac{\partial N}{\partial y}(\xi, y)| \sup_{x\in[0,1]} |v''(x)|).$$

Note that N is a piecewise linear function of ξ and so $\frac{\partial N}{\partial y}$ is also piecewise linear. So,

$$\sup_{\xi\in[0,1], y\in[-2(\lambda+1)I', 2(\lambda+1)I']} |\frac{\partial N}{\partial y}(\xi, y)| \leq 2 \sup_{y\in[-2(\lambda+1)I', 2(\lambda+1)I']} |\frac{\partial [N]}{\partial y}(y)|.$$

Function v'' can be evaluated using the relation corresponding to the derivative of (45). Taking into consideration these estimates we show that the order of estimate (11) is the same as in Theorem 3.

The homogenization of the cable equation is a more complicated problem, however if the homogenized cable equation

$$\frac{\partial}{\partial x}\Big(\langle\frac{1}{\sigma_\delta\Big(\xi,[N](v')\Big)}\rangle^{-1}\frac{\partial v}{\partial x}\Big)=\langle A_m C_m\rangle\frac{\partial v}{\partial t}+\langle A_m I_{ion}(v,\boldsymbol{w})\rangle\quad\text{in }\Omega\times(0,T),\tag{46}$$

$$\frac{\partial \boldsymbol{w}}{\partial t}=g(v,\boldsymbol{w}),\tag{47}$$

supplied with the conditions

$$v|_{t=0}=0,\boldsymbol{w}|_{t=0}=0,-\Big(\langle\frac{1}{\sigma_\delta\Big(\xi,[N](\frac{\partial v}{\partial x})\Big)}\rangle^{-1}\frac{\partial v}{\partial x}\Big)|_{x=0}=I_0,\tag{48}$$

admits a unique solution with bounded derivatives $\frac{\partial v}{\partial x},\frac{\partial v}{\partial t},\frac{\partial^2 v}{\partial x^2}$ for lipschitzian I_{ion} and $g(v,\mathbf{w})$ we can prove that $u^{(1)}_{\varepsilon,\delta}=v+\varepsilon N$ satisfies cable equation with a residual of order $O(\varepsilon+\delta)$, i.e. $u^{(1)}_{\varepsilon,\delta}$ is a first order formal asymptotic approximation.

Based on the macroscopic homogenized equations, we express the non-Ohmic cable problem of cardiac electrophysiology as follows: Find a macroscopic transmembrane potential function v such that

$$\frac{\partial}{\partial x}\big(\hat{\sigma}(\frac{\partial v}{dx})\frac{\partial v}{\partial x}\big)=A_{\mathrm{m}}\left\{C_{\mathrm{m}}\frac{\partial v}{\partial t}+I_{\mathrm{ion}}\right\}\quad\text{in }\Omega\times(0,T),\tag{49}$$

where $I_{\mathrm{ion}}:\mathbb{R}\times\mathbb{R}\to\mathbb{R}$ represents the transmembrane ionic current, C_{m} is the membrane capacity and A_{m} is the surface-to-volume ratio, and we note that the right-hand side of (49) accounts for the amount of charge that leaves the intracellular domain and enters the extracellular domain. Further, we will assume that the transmembrane ionic current I_{ion} is governed by v and by gating variables $\boldsymbol{w}:\Omega\times(0,T)\to\mathbb{R}^M$ that modulate the conductance of ion channels, pumps and exchangers, i.e., $I_{\mathrm{ion}}=I_{\mathrm{ion}}(v,\boldsymbol{w})$, where the exact functional form of I_{ion} will depend on the choice of ionic model. The evolution of gating variables is determined by kinetic equations of the form

$$\frac{\partial \boldsymbol{w}}{\partial t}=g(v,\boldsymbol{w}),\tag{50}$$

where the form of $g:\mathbb{R}\times\mathbb{R}^M\to\mathbb{R}^M$ will also depend on chosen the ionic model. The equations (49) and (50) are supplemented with initial and boundary conditions for the transmembrane potential and gating variables to form an initial boundary value problem.The homogenization of the cable equation with linear Ohm's law has been addressed elsewhere [6–8].

Remark 2. Applying some heuristic arguments (the principle of splitting of the homogenized operator, [1], p.246, see also [4] p. 316) we can formally generalize the macroscopic model to the three-dimensional case: assuming that the cytoplasm occupies the boxes $((k_1+\delta/2)\varepsilon,(k_1+1-\delta/2)\varepsilon)\times((k_2+\delta/2)\varepsilon,(k_2+1-\delta/2)\varepsilon)\times((k_3+\delta/2)\varepsilon,(k_3+1-\delta/2)\varepsilon)$, where k_i are integers, supposing that the gap junctions occupy the layers $\{x_i\in((k_i-\delta/2)\varepsilon,(k_i+\delta/2)\varepsilon)\},i=1,2,3$, and ignoring the conductivity in the intersection of the layers $\{x_i\in((k_i-\delta/2)\varepsilon,(k_i+\delta/2)\varepsilon)\}\cap\{x_j\in((k_j-\delta/2)\varepsilon,(k_j+\delta/2)\varepsilon)\},i\neq j$, we can rewrite equation (46) replacing the left hand side by

$$\sum_{i=1}^{3}\frac{\partial}{\partial x_i}\Big(\langle\frac{1}{\sigma_\delta\Big(\xi,[N](\frac{\partial v}{\partial x_i})\Big)}\rangle^{-1}\frac{\partial v}{\partial x_i}\Big)$$

In the case of anysotropy when a,μ,σ_c,σ_g depend on the direction ($a=a_i,\mu=\mu_i,\sigma_c=\sigma_{ci},\sigma_g=\sigma_{gi}$) then we get the left-hand side

$$\sum_{i=1}^{3}\frac{\partial}{\partial x_i}\Big(\hat{\sigma}_i(\frac{\partial v}{\partial x_i})\frac{\partial v}{\partial x_i}\Big)$$

where $\hat{\sigma}_i$ is constructed as $\langle \frac{1}{\sigma_\delta\left(\xi,[N](\frac{\partial v}{\partial x_i})\right)} \rangle^{-1}$ but with $a, \mu, \sigma_c, \sigma_g$ replaced by $a_i, \mu_i, \sigma_{ci}, \sigma_{gi}$ respectively.

2 Numerical solution

The homogenized equations were numerically solved using an explicit Euler finite-element spatio-temporal discretization scheme. To this the, we start by defining the trial and test spaces:

$$\mathcal{S} = \left\{ v \in (L^2(0,T]; H^1(\Omega, \mathbb{R})) \right\}$$
$$\mathcal{V} = \left\{ \nu \in H^1(\Omega, \mathbb{R}) \right\}$$

Testing the function $\nu \in \mathcal{V}$ against (49) we obtain the the weak form of the non-Ohmic electrophysiology which reads: Find $v \in \mathcal{S}$ such that

$$\int_\Omega A_\mathrm{m} C_\mathrm{m} \frac{\partial v}{\partial t} \nu \, dx + \int_\Omega \frac{\partial \nu}{\partial x} \hat{\sigma} \left(\frac{\partial v}{\partial x} \right) \frac{\partial v}{\partial x} \, dx + \int_\Omega A_\mathrm{m} I_\mathrm{ion}(v, \boldsymbol{w}) \nu \, dx + \bar{j}_0 \nu(0) + \bar{j}_L \nu(L) = 0, \quad \forall \, \nu \in \mathcal{V}.$$

were $\bar{j}_0$ and $\bar{j}_L$ are the flux applied in the left and right end respectively. For all simulations we have $\bar{j}_0 \neq 0$ and $\bar{j}_L = 0$ except when we elicit the electrical wave from right to left. In this case $\bar{j}_0 = 0$ and $\bar{j}_L \neq 0$. Using a Forward-Euler explicit scheme to discretize in time assuming a uniform partition $[0, \dots, t_n, t_{n+1}, \dots, T]$, where the time step is defined by $\Delta t = t_{n+1} - t_n$. Then, given v_n and $\boldsymbol{w}_n$, we find v_{n+1} from the semi-discrete equation

$$\int_\Omega A_\mathrm{m} C_\mathrm{m} \frac{v_{n+1} \nu}{\Delta t} \, dx - \int_\Omega A_\mathrm{m} C_\mathrm{m} \frac{v_n \nu}{\Delta t} \, dx + \int_\Omega \frac{\partial \nu}{\partial x} \hat{\sigma} \left(\frac{\partial v_n}{\partial x} \right) \frac{\partial v_n}{\partial x} \, dx + \int_\Omega A_\mathrm{m} I_\mathrm{ion}(v_n, \boldsymbol{w}_n) \nu \, dx + \bar{j} \nu(0) = 0,$$

which we write in abstract variational form as: find v_{n+1} such that

$$a(v_{n+1}, \nu) = F(\nu) \quad \forall \, \nu \in \mathcal{V} \tag{51}$$

where

$$a(v, \nu) = \int_\Omega A_\mathrm{m} C_\mathrm{m} \frac{v_{n+1} \nu}{\Delta t} \, dx$$
$$F(\nu) = \int_\Omega A_\mathrm{m} \left\{ C_\mathrm{m} \frac{v_n \nu}{\Delta t} - I_\mathrm{ion}(v_n, \boldsymbol{w}_n) \right\} \nu \, dx - \int_\Omega \frac{\partial \nu}{\partial x} \hat{\sigma} \left(\frac{\partial v_n}{\partial x} \right) \frac{\partial v_n}{\partial x} \, dx - \bar{j} \nu(0).$$

The gating variables are updated locally using a forward Euler scheme, which results in the update

$$\boldsymbol{w}_{n+1} = \boldsymbol{w}_n + \Delta t \, g(v_n, \boldsymbol{w}_n). \tag{52}$$

The time-discretized variational problem described in (51) and the internal variable update (52) were implemented and solved in FEniCS (`https://fenicsproject.org`) using version 2018.1.0.

References

1. Bakhvalov N, Panasenko G. Homogenisation: Averaging Processes in Periodic Media. Dordrecht/Boston/London; 1989.

2. Bensoussan A, Lions JL, Papanicolaou G, Caughey TK. Asymptotic Analysis of Periodic Structures. Journal of Applied Mechanics. 1979;doi:10.1115/1.3424588.

3. Jikov VV, Kozlov SM, Oleinik OA. Homogenization of Differential Operators and Integral Functionals. Berlin-New York: Springer-Verlag; 1994.

4. Panasenko G. Multi-scale Modelling for Structures and Composites. Berlin/Heidelberg: Springer-Verlag; 2005. Available from: `http://link.springer.com/10.1007/1-4020-2982-9`.

5. Sanchez-Palencia E. Non-Homogeneous Media and Vibration Theory. New York: Springer-Verlag; 1980.

6. Neu JC, Krassowska W. Homogenization of syncytial tissues. Critical Reviews in Biomedical Engineering. 1993;21(2):137–199.

7. Pennacchio M, Savare G, Colli Franzone P. Multiscale modeling for the electrical activity of the heart. SIAM J Math Anal. 2006;37(4):1333–1370.

8. Hand PE, Griffith BE, Peskin CS. Deriving macroscopic myocardial conductivities by homogenization of microscopic models. Bulletin of Mathematical Biology. 2009;71(7):1707–1726. doi:10.1007/s11538-009-9421-y.
