# Supplementary material for: Non-ohmic tissue conduction in cardiac electrophysiology: Upscaling the non-linear voltage-dependent conductance of gap junctions
Source: PLoS Comput Biol. 2020 Feb 25;16(2):e1007232. doi: 10.1371/journal.pcbi.1007232 (PMC7059938; doi:10.1371/journal.pcbi.1007232)
Supplement: Attachment — Submitted filename: report.pdf [file pcbi.1007232.s002.pdf]

Ref-report on Non-ohmic tissue conduction in cardiac electrophysiology - upscaling the non-linear voltage-dependent conductance of gap junctions by D. E. Hurtado, J. Jilberto, and G. Panasenko

Most tissue-level models of cardiac electrophysiology make use of continuum approximations to discrete cell-level equations. A commonly used model is based on the monodomain (or bidomain) reaction-diffusion equations, which follows from a homogenization process. However, as is well known, this derivation does a poor job accounting for the presence of gap junctions that connect one cell to another. Experimental results show that gap junctions can have a significant effect on the propagation of the action potential. Several works have been devoted to improving this aspect of the mondomain/bidomain model. Linear-gradient constitutive laws are the basis for most models in continuum mechanics and biology (linear Ohm law). In the paper under review the authors incorporate gap junction structures into the microscopic (cell-level) equations using a "nonlinear Ohm law" that regulate the current by a conductivity that depends nonlinearly on the so-called transjunctional potential jump (the relative difference in the membrane potential between coupled cells). After a simple and formal homogenization process the authors obtain a model in which the elliptic (diffusion) part contains a gradient-dependent conductivity. Moreover, the conductivity function itself is implicitly defined via an algebraic equation. The main part of the paper is devoted to presenting and testing (numerically) this model.

In addition, there is a "mathematical" appendix that derives the model in a very simplified setting. Let us provide some details regarding this derivation. The authors consider conduction in a strand of cells $\Omega = (0,1) \subset \mathbb{R}$, where the cell length is ε and the length of gap junctions is $\varepsilon\delta$, with $\varepsilon\delta$ much smaller than ε and ε much smaller than 1. In other words, δ is an additional (small) parameter determining the size of gap junctions relative to cell length. The cytoplasma is represented by a union of intervals of the form

$$I_j^c := \big((j+\delta/2)\varepsilon, (j+1-\delta/2)\varepsilon\big), \qquad j \in \mathbb{Z},$$

of length $\varepsilon(1-\delta)$, while the gap junctions are represented by a union of intervals of the form

$$I_j^g := \big((j-\delta/2)\varepsilon, (j+\delta/2)\varepsilon\big), \qquad j \in \mathbb{Z},$$

of length $\varepsilon\delta$. See the figure on the right (Fig. 5 in the paper) for an illustration. The microscopic transmembrane potential and current are denoted respectively by u_ε and j_ε. The steady-state current balance reads

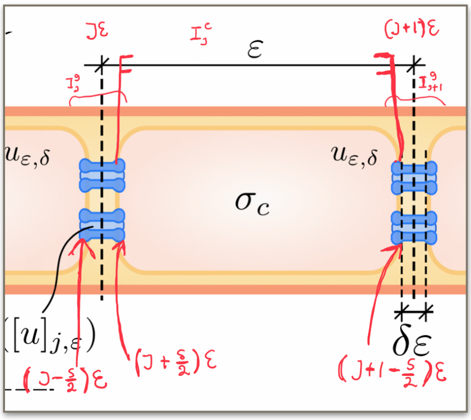

$$-\partial_x j_\varepsilon = 0,$$

where the basic assumption is that j_ε is of the form

$$j_\varepsilon = -\,\sigma\left(\frac{x}{\varepsilon}, u_\varepsilon(\,\cdot\,)\right)\partial_x u_\varepsilon.$$

The authors postulate that the conductivity σ takes the specific form

$$\sigma\left(y,u(\cdot)\right)=\begin{cases}\sigma_c, & y\in I_j^c/\varepsilon\\ \delta\,\sigma_g\left(1+\mu\,a\left([u]_{j,\varepsilon}\right)\right), & y\in I_j^g/\varepsilon\end{cases},$$

where σ_g, μ are constants, a is a bounded function, and

$$\varepsilon\,[u]_{j,\varepsilon}=u\left(\left(j+\frac{\delta}{2}\right)\varepsilon\right)-u\left(\left(j-\frac{\delta}{2}\right)\varepsilon\right)$$

denotes the jump of the function u accross the jth gap junction. In other words, the current follows the usual linear Ohm law inside the cytoplasma with conductivity σ_c, while at gap junctions the current is nonlinearly controlled by the function a and its dependency on the so-called transjunctional potential jump $[u]_{j,\varepsilon}$. From the point of view of standard homogenisation of 1D elliptic equations, the principal difficulty is that the discontinuous conductivity depends nonlinearly on the transjunctional potential jump. The authors claim that the homgenized problem (obtained by sending $\varepsilon\to 0$) is a nonlinear elliptic equation with gradient-dependent conductivity $\overline{\sigma}$, namely

$$-\partial_x\left(\overline{\sigma}(\partial_x v)\partial_x v\right)=0\quad\text{in } (0,1),$$

with boundary conditions

$$\overline{\sigma}(\partial_x v)\partial_x v\Big|_{x=0}=I_0\in\mathbb{R},\qquad v\,|_{x=0}=0.$$

The averaged conductivity $\overline{\sigma}=\overline{\sigma}(\xi)$ is defined by

$$\overline{\sigma}(\xi)=\left\langle\frac{1}{\sigma(\,\cdot\,,J(\xi))}\right\rangle^{-1}=\left(\int_0^1\frac{1}{\sigma(y,J(\xi))}\,dy\right)^{-1},$$

where $J=J(\xi)$ solves the implicit equation

$$J=-(1-\delta)\left(\left\langle\frac{1}{\sigma(\,\cdot\,,J)}\right\rangle^{-1}-1\right)\xi.$$

The above homogenized equation is derived in a very simplified context. It does not really model electrical conduction in cardiac biological tissues. However, the authors postulate (without further justification) that the standard monodomain reaction-diffusion equation should be modified to account for nonlinear conduction by replacing the linear elliptic operator $\partial_x\left(\sigma(x)\partial_x v\right)$ by the nonlinear elliptic operator $\partial_x\left(\overline{\sigma}(\partial_x v)\partial_x v\right)$ that depends on the gradient of the potential. It is this model that is utilized in the numerical examples.

Overall, the *first part* of paper is carefully written and makes for a very pleasant read. It contains interesting results that attempt to rectify some of the deficiencies (related to gap junctions) of the commonly used models for electrical conduction in biological tissues.

The second part of the paper (appendix), which is is devoted to the derivation of the homgenized model, is poorly written and needs to be largely reworked. The organization is not clear. In fact, it is very difficult to read because of typos and inaccuracies in notation as well as in the "mathematics". It is hard to detect a logical structure in the presentation. For example, the homogenized

conductivity coefficient is implicitly defined. It solves an "algebraic" equation. It would be natural to discuss early on in the paper that this equation is well-defined and can indeed be solved to find the conductivity. This is not done as far as I can see. Instead it is hidden in a few sentences (discussing fixed points) at the very end of the paper. This does not make for easy reading. By the way, the notation makes it somewhat difficult to understand the precise definition of the homogenized conductivity function $\hat{\sigma}_\delta$. Perhaps after defining this function you include an example where $\hat{\sigma}_\delta$ is explicitly computed for a simple choice of the function a. By the way, is it correct to insert an ε in $[u]_{j,\varepsilon}$ in eqn. (4), appendix? It is not clear what the authors mean by a solution to their PDEs. Is it a weak solution or a classical solution. A classical solution demands that the solution is twice continuously differentable. I found no arguments showing that the involved functions actually possess this regularity. Is it available? I recommend that the authors add references to classic homogenization theories. Homogenization of transmission problems with interface jumps can be found in numerous works concerning models of diffusion in various applications. Discuss and relate your arguments to revelant existing works. Be more precise when you define the functional spaces, in particular those that involve periodic functions. Spaces of periodic functions often use the subscript #. It is difficult at times to understand if functions are periodically extended to the entire domain or simply defined on an intervall I_j^c, I_c^g. Increase the overall precision when presenting the mathematics ... The role of the parameter $\delta > 0$ is unclear. The homogenization parameter ε vanishes in the macroscopic model? What about δ? Several places in the manuscript the functions carry the subscripts ε, δ but the macrospic model seems to depend on δ via the “averaged” conduction coefficient. If δ is a fixed number, why indicate that the functions depend on δ? This is confusing. Adding to this confusion, corrector error estimates (eq. 12) seem to depend δ (not ε). In eqn. (26) you have neglected the term $\delta v'((\xi + \theta\delta)\varepsilon)$ (define θ!) from eqn. (24). Justify why you can do that, i.e., is not possible that $\delta v'(\cdots) = \mathcal{O}(1)$? I do not understand Theorem 2, in particular the hypotheses. As a final remark, I believe that the paper would have benefitted from writing out a detailed two-scale homogenization argument for the monodomain equation (instead of the very simplied 1D elliptic setting chosen by the authors); after all, this is the model used in numerical simulations.

To summarize: I have listed a few comments/remarks regarding the appendix but there are numerous others that I do not list. Overall, the writing of the appendix must be significantly improved before this paper can be accepted for publication.
