# Supplementary material for: Non-ohmic tissue conduction in cardiac electrophysiology: Upscaling the non-linear voltage-dependent conductance of gap junctions
Source: PLoS Comput Biol. 2020 Feb 25;16(2):e1007232. doi: 10.1371/journal.pcbi.1007232 (PMC7059938; doi:10.1371/journal.pcbi.1007232)
Supplement: Attachment — Submitted filename: ResponseLetter_R01.pdf [file pcbi.1007232.s003.pdf]

Response Letter to Reviewers – Manuscript PCOMPBIOL-D-19-01074 "Non-ohmic tissue conduction in cardiac electrophysiology: upscaling the non-linear voltage-dependent conductance of gap junctions"

We thank the editors and reviewers for the constructive feedback and comments, which have help us to improve our work. The manuscript has been revised accordingly, with changes marked in red. Below you will find a point-by-point answer letter to the concerns raised.

Reviewer 1

The study by Hurtado et al presents a cardiac tissue model that accounts for nonlinearity of gap junction conductance, specifically deriving and presenting the numerical solution of a homogenized model that incorporates voltage-dependent gap junctions. As noted by the authors, non-ohmic dynamics is an under-appreciated aspect of gap junction behavior that is typically not accounted for in many cardiac tissue models. This study presents a nice potential approach to account for these details without a significant increase in computational complexity.

However, there are several major issues for the authors to address:
- The biophysical basis for the non-Ohmic behavior is a consequence of the voltage-dependent gating of the gap junction hemichannels (similar to the voltage-dependent gating of other sarcolemmal ion channels). My most significant concern is that the model formulation appears to neglect a critical aspect of the gating behavior, in particular that the gap junction conductance, in addition to being a function of transjunctional voltage (Vj), is also time-dependent. That is, the gating of the gap junction protein hemichannels has a time-dependence that also depends on Vj. This is demonstrated in a wide range of studies, see for example work from Weingart (including ref 3), Veenstra, Bukauskas, Bennett, and many others.

The time constant for changes in gap junction conductance is generally found to be a decaying function of the Vj magnitude, with values on the order of a few seconds when Vj = 0 mV. As presented, the model formulation here appears to assume that the gating of the gap junctions is instantaneous. This is problematic because, in the model, gap junction conductance changes are thus much faster than the dynamics of sarcolemmal ion channels, whereas physiologically, gap junction conductance changes are much slower.

When cells are well coupled, the absolute value of Vj is generally not much greater than 0 mV for durations on the order of at most 10s of milliseconds, and thus gap junction conductance generally does not approach the reduced steady-state levels shown at the extreme Vj values in Figure 3.

However, when cells are poorly coupled, i.e., low baseline gap junction conductance levels, large Vj values can occur for longer durations, which in turn does result in transient decreases in gap junction conductance. This has been previously demonstrated by Henriquez et al (ref 20 in the manuscript), and more recently by Weinberg (Chaos, 2017). At a minimum, the authors should compare their work with these prior studies, which are two of the few studies that have accounted for non-linear gap junction conductance in a tissue model, with Weinberg also including electrical field coupling.

The lack of accounting for the time-dependence of gap junction conductance changes is a significant limitation that detracts from potential impact of the study. However, perhaps this can

be incorporated into the proposed framework by including additional gating variables into the w gating variable vector, following approaches similar to either the Henriquez et al or Weinberg studies noted above, which both include time-dependent gap junction conductance changes.

A: Thank you for these very important comments. We absolutely agree that the time dependence of gap-junction (GJ) conduction is indeed a major determinant of intercellular communication, as remarked by the reviewer. Not including this dependence in our model is certainly an important limitation that we now thoroughly discuss in detail in the discussion of the revised manuscript. The main reason at this point for not including time evolution in the GJ conduction model is the fact that homogenization theory works well for upscaling spatial fine-scale variations but has not been fully explored/developed for upscaling fine-scale time variations, which necessitates further highly-technical arguments that are outside the scope of this contribution. Having said that, and taking into consideration the reviewer's comments, we now distinguish and study two limit cases for the time-dependent conduction of GJ: the i) instantaneous conductance, and the ii) steady-state conductance. Simulations of wavefronts, conduction velocity for a range of different levels of gap-junctional coupling, and restitution curves for these two limiting cases are now included in the manuscript, and we discuss in detail the differences. Very interesting conclusions arise from the consideration of these two limiting cases, please find them in the revised manuscript.

We appreciate the suggestion of the reference Weinberg (Chaos 2017), which we were not aware of. It has been included in the manuscript, and we now compare some of our results with those included in that work and in the work of Henriquez et al. (2001).

- My other significant concern is the lack of description of the cell-chain model from Kucera et al (ref 5) that is considered the "baseline" in this study. The paper by Kucera and colleagues described two variants of their model, so it is not clear which version of the Kucera et al model is used and what are the associated parameters. In particular, in addition to discretizing each cell into membrane patches of 10 um (as the authors note), Kucera et al also describe a "non-cleft" and "cleft" version of the model, in which electric field coupling occurs via extracellular current in the intercellular cleft in the "cleft" version. Additionally, Kucera et al study the significance of redistributing the voltage-gated sodium current from axial to intercalated disk membrane patches and variations in intercellular cleft width. (The authors note this redistribution of sodium channels in the Discussion but not in the description of the baseline model.)

Since the focus of the Kucera et al paper is the "cleft" version, one would assume that this version of the model was used in the current study, but this needs to be clarified. Regardless of which version of the Kucera et al model is used, both versions of the model include a "Rgap" term – a constant gap junction resistance between cells. It is not clear if the non-ohmic conductance is also incorporated into the baseline model, or if Rgap is a constant in the baseline model.

Similarly, the linear homogenized model that is compared (LHM) from Hand and Peskin (ref 12) also incorporates intercellular cleft electric field coupling and sodium channel distributions. However, the authors describe this model as a "standard cable model." This is a confusing

description, because the classical description of the cable model or the monodomain model does not include electric field coupling and assumes uniform distribution of sodium channels. A significantly more detailed description of these models used for comparison is needed.

A: Thank you for these comments, which are also shared by Reviewer 2. The revised manuscript now includes in the Methods section a detailed description of two cellular models (CM): i) the CM voltage-gated, which considers a nonlinear resistor between adjacent cells representing the voltage-dependent conductance of gap junctions, and ii) the CM clamped, which assumes a constant (voltage-insensitive) conductance to represent gap junctions. We also included in the revised manuscript how the LHM is constructed from the NOHM by assuming a constant conductivity for gap junctions. We have limited (but not removed) the references to the Kucera and Peskin models to avoid confusion, as they focus on the ephatic effect, which is not the focus of our work.

The reason for including two cellular models was to be more consistent in comparing cellular versus continuum models of propagation: for the voltage-dependent case of gap junctions, the NOHM was contrasted with the CM voltage-gated as its baseline. For the case of voltage-insensitive gap junctions, the LHM was contrasted with the CM clamped as its baseline. In this way, we distinguish between truly non-Ohmic (voltage-dependent) and purely Ohmic (voltage-insensitive) cases of conduction. Very interesting results arise from these models and their comparisons, which are now included in the manuscript.

- The authors should expand significantly on the reasons why the non-ohmic model and the baseline model agree in some parameter regimes (specifically higher coupling) and disagree in other regimes (weaker coupling). In the regimes where the models disagree, then presumably there are some assumptions of the derivation that fail, such that the homogenization is not valid. These are important limitations that the authors should comment on and discuss, especially since, as the authors note, that slower conduction is often pro-arrhythmic and is thus of significant interest in simulations.

A: Comparisons of models and the associated discussion and conclusions have been revised according to our point above. We now show in Figure 5 of the revised manuscript that continuum models, in general, do approximate their cellular counterparts reasonably well for a wide range of gap-junctional coupling levels. Interestingly, for the steady-state regime of conductance, voltage-dependent models (CM voltage-gated and NOHM) show important deviations from voltage-insensitive models (CM clamped and LHM), which highlights the importance of modeling gap-junction voltage dependence under steady-state regimes of conduction. Further, we show that the NOHM nicely captures conduction block at low coupling, a feature that the LHM does not capture. These new results are thoroughly documented and discussed in the revised manuscript.

- What is the baseline gap junction conductance value associated with the simulations in Figure 4? Based on the conduction velocity values, it appears the cells are well coupled, similar to as in Figure 1

A: Nominal (representative) gap junction conductance values are now included in the revised manuscript. They are the same values reported in Kucera et al (2007). The level of coupling is now handled through the parameter beta.

In this well-coupled case, a conduction velocity of 50 cm/s implies that propagation of a distance of 100 um will take 0.2 ms, which is less than the duration of the cardiac action potential upstroke, so Vj magnitudes are probably on the order of 10-20 mV at most. Even without accounting for the time-dependence of the gap junction conductance as noted in comment 1, based on the curves in Figure 3, steady-state conductance levels are within 20% of the baseline value, so it is surprising that conduction velocity values differ by nearly 50%. Can the authors explain this result by examining the Vj curves and associated changes in gap junction conductance along the cable?

A: Thank you for this comment. Figure 5 of the revised manuscript shows the CV for homotypic channels. For the instantaneous conduction regime, no differences in the CV were found between the Cx43-Cx43 and the Cx45-Cx45 channels at full coupling. For the steady-state case, the CV for the Cx43-Cx43 was roughly 64 cm/s, and the CV for the Cx45-Cx45 was 48 cm/s at full coupling. So CV values between these two channels differ by 25%. This difference in CV is consistent with the fact that, for a transjunctional voltage of Vj=20 mV, the normalized conductance for Cx43-Cx43 is roughly 1.0, while the normalized conductance for Cx45-Cx45 is roughly 0.8, giving a difference of around 20%.

In the case of the heterotypic channel Cx43-Cx45, Figure 6 of the revised manuscript shows that at full coupling under steady-state conductance, CV is roughly 24 cm/s in normal (left-to-right) propagation and 68 cm/s in retrograde propagation, giving a difference of 65%. Looking at the conductance distribution for this channel (Figure 3(c)) at steady-state, normal propagation at Vj=-20 mV yields a normalized conductance of 0.4, whereas retrograde propagation at Vj=20 mV yields a normalized conductance of roughly 1.2, i.e., giving a difference of roughly 65% too. In conclusion, normalized conductances at the levels of transjunctional voltage expected for full coupling do result in consistent CVs.

- The differences in conduction velocity for different directions shown for the heterotypic gap junction, illustrated in Fig 4C, is one of the more interesting results of the paper. However, this point is demonstrated for a single case (i.e., one unknown value of gap junction coupling, see previous comment), and thus it is not clear for what conditions these directional differences are small or large. For example, are there conditions in which propagation fails in one direction but not the other? The authors should show a plot similar to Fig 2A plotting conduction velocity for both directions for different conductance levels.

A: Thank you for this valuable suggestion. We have simulated the heterotypic GJ case for

varying levels of Gj coupling, normal and retrograde propagation, and for all four conduction models, please see Figure 6 in the revised manuscript. One remarkable result is that, for steady-state regimes, conduction block is orientation dependent (Figure 6, left) and occurs at a GJ coupling level of 10%. This phenomenon predicted by the CM voltage-gated model is nicely captured by the NOHM, but not by the LHM.

It would also be interesting to study if there is a pacing rate dependence. For example, are there conditions in which conduction in both directions is similar at slow pacing rates, but differs for faster pacing rates?

A: Based on this comment, we studied the pacing rate dependence in the heterotypic Cx43-Cx45 channel by simulating and constructing CV restitution curves, please see Figure 7 in the revised manuscript. CV is orientation-dependent for the whole range of pacing rates studied and presents a uniform shift towards greater CV values under retrograde propagation.

- The derivation of the model shown in the Appendix is fairly difficult to follow. An important contribution would be specifically highlighting how this derivation differs from the homogenization required for such a model in which gap junction conductance is constant.

A: We have expanded the NOHM and LHM model description in the manuscript, showing how the LHM can be obtained as a particular case of the NOHM with constant gap-junction conductance, and left the more technical details in the appendix.

- The sentence beginning with "Alternatively, …" at line 48 is an incomplete sentence.

A: Corrected, thank you.

- Line 67, "an" should be "and"

A: Corrected, thank you.

- In Fig. 4, the conduction velocity for the Cx43-Cx45 gap junction model is given as 32.1 cm/s in panel A and then 32.2 cm/s in panel B. Is this a typo since – as I understand it – these are referring to the same simulation condition?

A: It was a typo, now Figure 4 has changed. Thank you.

Reviewer 2

- The paper describes the development of a macroscopic tissue model for electrical conduction in cardiac tissue, which incorporates non-linear voltage-dependent conduction through gap junctions. The topic is important and relevant for the research community in computational cardiac electrophysiology, and the paper presents a new modeling approach that could potentially have significant implications. However, I have some concerns related to the model derivation and the discussion of the results, which should be improved before publication. Furthermore, although the manuscript is generally well written, the overall structure and ordering (Results-Discussion-Methods) makes it somewhat hard to read. I assume the structure is dictated by the journal, which raises the question of whether PLOS Computational Biology is the best target for this fairly mathematical and model-oriented manuscript.

A: We agree with the reviewer that this work is best understood by reading the 'Methods' section before the 'Results' section. According to this suggestion, the structure of the manuscript has been reordered to Methods-Results-Discussion. PLOS allows for such change as long as it is justified, which we agree in this case it is.

Major concerns:

- The model derivation described in the Methods section is not based on physically meaningful properties. In appendix S1 the homogenization is performed in terms a generic microscopic potential and microscopic current density, which in this context must be interpreted as intracellular properties. However, in eq (3) in the Methods section the intracellular/GJ current density is computed by multiplying the transmembrane potential with the cytoplasm/GJ conductivity. This is only correct if the extracellular potential is constant. If this is assumed it should be mentioned explicitly in the derivation, since it is a significant limitation with potential implications for the model's range of validity. I would recommend that the model derivation is based explicitly on balance of intra- and extracellular currents, expressed in terms of intra- and extracellular potentials, and that all assumptions leading to the final model are made explicit. It should also be considered if a more generic 2D/3D version of the model could be derived, since the restriction to 1D is a severe limitation.

A: This is a very relevant comment, thank you. Our work does assume that the extracellular potential is constant, which allow us to use the transmembrane potential in modeling the intracellular conduction. The revised manuscript now explicitly mentions this assumption right after Eqn. (1). For the sake of simplicity, we prefer to keep the transmembrane potential as the main unknown field, as it facilitates the development of the mathematical theory of non-linear homogenization and merge it with the transmembrane ionic currents in a single balance equation. We have also explicitly acknowledged and discussed this limitation in the Discussion section of the revised manuscript.

While we also agree that the restriction to 1D conduction can be an important limitation, which was originally mentioned in the Discussion section of the manuscript, we remark that virtually all

of the microscopic studies of cardiac non-Ohmic conduction reported to date have been developed considering only 1D models of cell networks. Therefore, in order to be able to validate our model and compare our results with previously published data, the 1D setting seems the most reasonable one to generate an interesting discussion, and the one that can be easily interpreted and analyzed. We look forward to extending this framework to 3D in future contributions.

- The discussion of the results in relation to existing models is very limited. The GJ conduction models used by the authors seem well justified, and show interesting (although not entirely surprising) effects on conduction velocity. However, there are several alternative formulations of GJ conductance, including a variety of non-Ohmic and voltage-gated formulations. A comprehensive review of all existing models is obviously beyond the scope of the paper, but I would like to see a more thorough discussion of the results in the context of existing literature.

A: We thank the reviewer for this comment. We have greatly reworked and expanded the Discussion section in the revised manuscript to analyze the results from the additional models and experiments considered in this new version and we made sure to compare our results with the existing literature we were able to find.

- The baseline model is very briefly described. Although this model is described in some detail in the cited reference [5], it would be useful to recapitulate the main equations of the model in the present manuscript, or in a supplement. This would make the similarities and differences between the two models more apparent, and highlight relations between the model's parameters. In particular, it is not clear whether non-Ohmic GJ conduction is used for the baseline model, or if the original Ohmic formulation from [5] is used. Furthermore, it would be interesting to see the effect of discretization parameters both for the baseline model and the homogenized model. Does the conduction velocity of the baseline model change if the number of nodes per cell is increased or reduced? And what about the discretization of the NOM and LHM models?

A: Thank you for these comments, many of which were also raised by Reviewer 2 – please see our full answer above. In brief, the derivation of cellular models is now included in full detail in the manuscript, and we now consider the cases of constant and voltage-dependent conductance of gap junctions as baselines for the continuum models LHM and NOHM, respectively. Further, the effect of discretization both in cellular models and continuum models (with and without voltage dependence) is now reported in Figure 8. Yes, conduction velocity rapidly increases as the number of segments per cell is decreased. Interestingly, the conduction velocity in cellular models displays a mesh dependence that is much stronger than that observed in continuum models, which had been reported in the literature.

Minor issues:

- One page 3, lines 16-20, the discussion of existing literature could be more precise. The main topic of reference [5] is the study of sodium channel distribution related to GJs, which is not addressed in the present paper, and not to the GJ conduction itself.

A: Thank you, the reference was changed to another more relevant to gap-junction mediated communication.

- Page 3, lines 30-34: The formulation suggests that the monodomain model is based on the assumption of isotropic conductivity, which is not the case. Also, the most relevant model to reference in this context would be the bidomain model, since this is considered the most accurate model of cardiac electrophysiology, but is also based on the Ohmic assumptions used for the cable equation.

A: The introduction has been revised accordingly, and to avoid confusion we have eliminated the case of isotropic conductivity. We also introduce the bidomain model as the most complete one, with the monodomain representing a simplification.

- Page 4, lines 48-51: The sentence is incomplete.

A: Corrected, thank you.

- Page 4, lines 63-65: I assume the current referred to is a transmembrane current, but it would be useful to make this explicit.

A: We now indicate that excitation is due to an applied transmembrane current, thank you.

- Page 5, line 68: Is the LHM model the same model that would be obtained from inserting Ohmic GJc in the homogenization applied in this paper? If so, it could be useful to formulate it in this way, to make the model formulation and parameter specification more precise.

A: Yes, the LHM is a particular case of the NOHM when GJ conductance distribution is uniform. We now mention this in the manuscript, and the Appendix includes this calculation.

- On page 5, lines 74-79, it would improve readability if the change of GJc was explicitly referring to model parameter, stating which parameters are changed in the three models (LHM, NOM, baseline).

A: This is a good suggestion, thank you. The GJc is now parameterized in terms of beta, a parameter which is introduced in eqn. (5) of the revised manuscript. Figures have been revised to include this parameter in the modulation of GJc.

- Page 8, lines 142-143: Although capturing the low-conductance behavior is a strong feature of the proposed model, I would not describe the result as “remarkable”. As far as I can tell, all the proposed GJc models tested in the paper effectively shut down conduction as the voltage difference becomes large. Since low GJc will lead to increased cell-to-cell voltage difference, it is quite intuitive that the proposed models give conduction slowing compared with an Ohmic model. This could be commented on in the discussion.

A: Thank you for this suggestion. The Discussion section has been largely revised accordingly to include these observations.

- Page 10, eq (4): Why is the voltage jump divided by the cell length? (And there seems to be a mix of subscripts j and k)

A: In the revised manuscript, the voltage jump is no longer divided, please see eqn. (4).

- Page 11, line 211: Why is the ionic current a mapping from (R x R) to R?

A: It should be (R x R^M) to R, as the ionic current is governed by the transmembrane potential ‘v’ and gating variables ‘w’. It has been corrected in the revised manuscript, thank you.

- Page 11, line 227: The authors are to be applauded for intending to make all codes available for download. However, the listed github-repository is empty.

A: Codes have been uploaded to the GitHub, thank you.

Reviewer 3

- Overall, the first part of paper is carefully written and makes for a very pleasant read. It contains interesting results that attempt to rectify some of the deficiencies (related to gap junctions) of the commonly used models for electrical conduction in biological tissues. The second part of the paper (appendix), which is is devoted to the derivation of the homogenized model, is poorly written and needs to be largely reworked. The organization is not clear. In fact, it is very difficult to read because of typos and inaccuracies in notation as well as in the "mathematics". It is hard to detect a logical structure in the presentation.

A: The appendix has been significantly revised. An introduction explaining the content of the Appendix and its structure has been included. We further added subsections in order to clarify what is done at each step. We hope that now the Appendix becomes more accessible to a broader scientific audience.

- The homogenized conductivity coefficient is implicitly defined. It solves an "algebraic" equation. It would be natural to discuss early on in the paper that this equation is well-defined and can indeed be solved to find the conductivity. This is not done as far as I can see. Instead it is hidden in a few sentences (discussing fixed points) at the very end of the paper. This does not make for easy reading.

A: In the presentation of the algorithm for the calculation of the macroscopic nonlinear Ohm's law of the revised appe we have added a comment concerning the existence and uniqueness of the solution of the implicit equation, so that the algorithm can be implemented.

- By the way, the notation makes it somewhat difficult to understand the precise definition of the homogenized conductivity function σ δˆ . Perhaps after defining this function you include an example where σ δˆis explicitly computed for a simple choice of the function a.

A: Thank you for this suggestion. We now include the simple case of Ohmic conduction as an example in the appendix, which delivers the standard result in linear homogenization theory.

- By the way, is it correct to insert an ε in [u] j,ε in eqn. (4), appendix?

A: We removed the small parameter in the denominator of the relation between the conductivity of the membrane and the voltage gap and introduced a new parameter S which may be finite or big (a scaling factor) and we modified all considerations with respect to this more realistic assumption.

- It is not clear what the authors mean by a solution to their PDEs. Is it a weak solution or a classical solution. A classical solution demands that the solution is twice continuously

differentable. I found no arguments showing that the involved functions actually possess this regularity. Is it available?

It is now explained that we consider always the classical solutions, and for the regularity assumptions on the coefficients they are equivalent to the weak formulations. By the way, in our case the classical solution of problem (1), (2) is a piecewise linear function.

- I recommend that the authors add references to classic homogenization theories. Homogenization of transmission problems with interface jumps can be found in numerous works concerning models of diffusion in various applications. Discuss and relate your arguments to relevant existing works.

A: We added the references to the classical books on the homogenization theory. We compared the homogenization procedures for the linear and nonlinear microscopic Ohm's law settings.

- Be more precise when you define the functional spaces, in particular those that involve periodic functions. Spaces of periodic functions often use the subscript # . It is difficult at times to understand if functions are periodically extended to the entire domain or simply defined on an interval I_j^c, I_c^g. Increase the overall precision when presenting the mathematics.

A: For the cell problem we introduced an appropriate Sobolev space of periodic functions.

- The role of the parameter $\delta > 0$ is unclear. The homogenization parameter ε vanishes in the macroscopic model? What about δ ? Several places in the manuscript the functions carry the subscripts ε, δ but the macroscopic model seems to depend on δ via the "averaged" conduction coefficient. If δ is a fixed number, why indicate that the functions depend on δ ? This is confusing. Adding to this confusion, corrector error estimates (eq. 12) seem to depend on δ (not ε).

A: Concerning the presence of two small parameters ε and δ we added an explanation that both parameters are independently small but we keep δ in the homogenized equation in order to make the error estimates more precise.

- In eqn. (26) you have neglected the term $\delta v'((\xi + \theta\delta)\varepsilon)$ (define θ !) from eqn. (24). Justify why you can do that, i.e., is not possible that $\delta v'(\cdots) = O(1)$? I do not understand Theorem 2, in particular the hypotheses.

A: We added the phrase that v is a bounded function so that δv is of order of 1δ. We added the hypotheses of Theorem 2.

- As a final remark, I believe that the paper would have benefitted from writing out a detailed two-scale homogenization argument for the monodomain equation (instead of the very simplied 1D elliptic setting chosen by the authors); after all, this is the model used in numerical simulations.

A: For the sake of accessibility of Appendix for a broad audience we believe that the simplest nonlinear microscopic model of the Ohm's law is an appropriate choice leaving the ”honest” homogenization of the cable equation with the gap depending conductivity for the future purely mathematical publication.

- To summarize: I have listed a few comments/remarks regarding the appendix but there are numerous others that I do not list. Overall, the writing of the appendix must be significantly improved before this paper can be accepted for publication.

A: The authors are grateful to the reviewers for their useful remarks. All new reworked pieces are written in red color.
