# Supplementary material for: Non-ohmic tissue conduction in cardiac electrophysiology: Upscaling the non-linear voltage-dependent conductance of gap junctions
Source: PLoS Comput Biol. 2020 Feb 25;16(2):e1007232. doi: 10.1371/journal.pcbi.1007232 (PMC7059938; doi:10.1371/journal.pcbi.1007232)
Supplement: Attachment — Submitted filename: ResponseLetter_R02.pdf [file pcbi.1007232.s004.pdf]

Response Letter to Reviewers – Manuscript PCOMPBIOL-D-19-01074R1 "Non-ohmic tissue conduction in cardiac electrophysiology: upscaling the non-linear voltage-dependent conductance of gap junctions"

We thank the editors and reviewers for the constructive feedback and comments, which have help us to improve our work. The manuscript has been revised accordingly, with changes marked in red. Below you will find a point-by-point answer letter to the concerns raised.

Reviewer #1:

The authors have mostly addressed my concerns. However, there are still a few issues that I would like the authors to address.

1. The inclusion of the instantaneous gap junction gating studies are a nice addition to the manuscript. The authors should more clearly describe the differences between the instantaneous and steady-state conductance levels. The authors simply state "Given the time-dependent behavior of the conductance of GJs …" This explanation is not sufficient for the typical reader of the journal.

Related to this, while the authors have made efforts to more clearly highlight the time dependence of gap junctional gating, there is still no mention of the time scale for this gating process, specifically that the time to reach steady state can be on the order of seconds, which is longer than the cardiac action potential and certainly longer than the action potential upstroke. The manuscript highlights the significant differences between results when assuming instantaneous vs steady-state gap junction conductance levels; however the manuscript needs to also clearly highlight that steady-state values are only likely reached in cases of poor coupling when large magnitude Vj values can persistent for more than milliseconds.

A: Thank you for this important comment. Following this suggestion, and to highlight the importance of the dynamic behavior in gap-junction conductance, we have added the following in the discussion section:

"To date, the time-dependent gating of gap junctions has been incorporated in a few cellular models of cardiac conduction [5,27], showing the importance of both voltage- and time-dependent dynamics. We note here that it takes several seconds for a gap-junction channel to reach steady-state conductance. Such a time scale can be much longer than the time window where transjunctional voltage is large during normal conduction, i.e., during action potential upstroke. Thus, during normal conduction, the steady-state regime is not expected to occur. In contrast, under cases of poor intercellular coupling, large transjunctional voltage can occur for longer periods, which potentially drive the gap-junction towards a steady-state conduction regime [27]."

2. It is still not clear to me why the paper from Kucera, Rohr, and Rudy (ref 21) is highlighted as cellular model for comparison. As previously commented, this paper describes two model versions, the "non-cleft" and "cleft" version, with the non-cleft version serving as the control or comparison. The focus of that paper is on the cleft version that considers preferential localization of sodium channels and electric field or ephaptic coupling in the intercellular cleft. At a minimum, the authors should describe the cellular model as the "non-cleft" model from Kucera, Rohr, and Rudy, which is identical to the model illustrated in Fig. 2 in the revised manuscript.
However, this paper is far from the first to investigate discontinuous propagation in cardiac tissue and discretize the cell into multiple compartment, as in the non-cleft model. There are several earlier papers from Plonsey, Henriquez, and Rudy (and likely many others) using similar models. Diaz, Rudy, and Plonsey, Annals of Biomed Eng, 1983; Henriquez and Plonsey, Med & Biol. Eng. & Comput, 1987; and Shaw and Rudy, Circ Res, 1997 are three such examples.

A: Thank you for this observation, and for the first suggested reference which we were not aware of. We have modified the manuscript and references to reflect that the cellular model comes from the works of Diaz et al. (1983) and Henriquez and Plonsey (1987).

3. The instantaneous gap junction conductance for the Cx43-Cx43 and Cx45-Cx45 pairs shown in Figure 3 do not appear to be symmetric with respect to Vj (although it is somewhat difficult to tell this by eye). Are these relationships asymmetric and if so, why? It is not obvious why the directionality should matter in homotypic channels.

A: The gap-junction conductance distributions for homotypic channels are slightly asymmetric, as the parameters for the positive and negative ranges of transjunctional voltage are slightly different, please see Table 1. These parameter values were directly taken from the literature, particularly from reference [3] of the manuscript where a fit to experimental conductance values is performed. These slight differences do not result in appreciable differences when running the reverse conduction experiments.

4. Is the shortest CL values in the CV restitution curves in Fig. 7 the shortest CL that elicited propagation in each case, or do all curves run to some value around 300 ms? If the later, simulations should be run for CL values down to the loss of propagation or capture for each case. This will demonstrate if there are differences between loss of propagation between the different cases. The CV restitution curves are typically much steeper for these short CL values, and thus there are likely to be much greater differences between the different cases. This is particular relevant to simulation of tachyarrhymias.

A: We were running simulations until a CL value of 320 ms. Now, we run simulations until the loss of propagation, see Figure 7 in the revised manuscript. The loss of propagation occurs for CL values between 310 to 330 ms, which is now mentioned in the manuscript. Both models, CM voltage-gated and NOHM lose propagation at similar CL values.

Minor:
1. For the results shown in Figures 4-6 and 8, what is the pacing cycle length used? This should be included in either the text or figure captions.

A: The pacing cycle length used in Figure 4-6 and 8 was CL=800 ms. This is now mentioned in the text of the manuscript.

Reviewer #2:

The authors have addressed all the concerns I had with the original manuscript.

A: Thank you.

Reviewer #3:

The revision is satisfactory.

A: Thank you.
